# Atopic heredity modifies the association between maternal vitamin D status in pregnancy and the risk of atopic disease in childhood: an observational study

**DOI:** 10.1186/s12937-022-00787-9

**Published:** 2022-05-17

**Authors:** Anna Amberntsson, Ellinor Carlson Kjellberg, Jenny van Odijk, Andrea Mikkelsen, Linnea Bärebring, Hanna Augustin

**Affiliations:** 1 Department of Internal Medicine and Clinical Nutrition, Sahlgrenska Academy, University of Gothenburg, Gothenburg, Sweden; 2 Department of Respiratory Medicine and Allergology, Sahlgrenska University Hospital, Gothenburg, Sweden; 3 Research and Development Primary Health Care, Gothenburg and Södra Bohuslän, Region Västra Götaland Sweden

**Keywords:** Vitamin D, Pregnancy, Neonatal outcomes, Atopic disease, Developmental origins of health and disease

## Abstract

**Background:**

The relationship between maternal vitamin D status in pregnancy and the development of atopic diseases in the offspring has been frequently studied, but with contradictory results. Previous studies have found an inverse relation between maternal vitamin D in pregnancy and the risk of atopic diseases in the child. In contrast, others have found a higher maternal 25OHD to be related to a higher risk of atopic diseases. Thus, the aim was to investigate the associations between maternal vitamin D status and intake in pregnancy with asthma, eczema and food allergies in the children up to 5 years. In addition, effect modification by reported atopic heredity was studied.

**Methods:**

Participants in the GraviD study had 25-hydroxyvitamin D (25OHD) analyzed in serum in early (T1) and late (T3) pregnancy. Maternal dietary vitamin D intake was estimated from a short food frequency questionnaire and supplement use by questionnaires. At 5 years of age the child´s history of asthma, eczema and food allergy, including atopic heredity, was reported by questionnaire. Multivariable logistic regression was used.

**Results:**

The cumulative incidence of asthma was 13%, eczema 22%, and food allergy 18%. Only among children without reported atopic heredity, maternal 25OHD of 50–75 nmol/L in T1 was associated with lower odds of asthma (OR 0.271, 95% CI 0.127–0.580), compared to maternal 25OHD > 75 nmol/L. Additionally in these children, maternal 25OHD in T3 (continuous) was associated with asthma (OR 1.014, 95% CI 1.002–1.009), and dietary vitamin D intake with eczema (OR 1.141, 95% CI 1.011–1.288).

**Conclusions:**

Among children without reported atopic heredity, higher maternal vitamin D status and intake during pregnancy was associated with increased risk of reported atopic disease.

**Supplementary Information:**

The online version contains supplementary material available at 10.1186/s12937-022-00787-9.

## Background

Atopic diseases are a considerable a burden both for the individual and its family, as well as for public health [1]. The reasons seem to be multi-etiological and involve e.g. environmental, genetic and nutritional factors [2]. In recent years, the relationship between maternal vitamin D status in pregnancy and the development of atopic diseases in the offspring has been more frequently studied, but the role of maternal vitamin D in atopic disease remains elusive [3–6].

Vitamin D is a fat-soluble vitamin, which is primarily produced in the skin after sun exposure and is also obtained via the diet [7]. Vitamin D status in pregnancy has been suggested to be part of the pathogenesis of atopic diseases in childhood, possibly through its involvement in the development of the fetal respiratory and immune system [5, 6, 8]. Some cohort studies have found an inverse relation between maternal vitamin D status (assessed as 25-hydroxyvitamin D, 25OHD) in pregnancy and the risk of developing asthma [4, 9] and eczema [9] in the offspring. Oppositely, others have found a higher maternal 25OHD to be related to a higher risk of atopic diseases [10, 11]. A Danish randomized controlled trial comparing high-dose to standard-dose of vitamin D (70 µg/day to 10 µg/day) during pregnancy found a non-significant protective effect of the high-dose of vitamin D on wheeze at 3 years of age [12], but no effect on either wheezing or asthma at 6 years of age [13]. A randomized controlled trial performed in the United States comparing vitamin D supplement of 10 µg/day to 110 µg/day in pregnant women found the incidence of asthma and recurrent wheezing at 3 years age was 6.1% lower but non-significant in the high-dose group [14]. Further, recent systematic reviews of both observational and interventional studies show heterogeneous results regarding maternal vitamin D status [15–17] and vitamin D intake [18–20] for prevention of atopic diseases in childhood. The inconclusive results of previous studies investigating maternal vitamin D in relation to atopic diseases might be affected by difference in time points of assessment of vitamin D status and intake during pregnancy, but also by not including the role of atopic heredity.

The aim of this study was to investigate the associations between maternal vitamin D status in early and late pregnancy, and vitamin D intake with the development of atopic diseases in the children up to 5 years age. In addition, the modifying effect of atopic heredity will be studied.

## Methods

### Study population

This paper includes data from the women participating in the Swedish GraviD study, and a follow-up of their children at 5 years of age. The GraviD study is a multi-ethnic longitudinal pregnancy cohort where women in early pregnancy with residency in south-western Sweden were recruited. Details on the procedure of the GraviD study are described elsewhere [21]. The only inclusion criteria was ≤ 16 gestational weeks. In total, recruitment during 2013 and 2014 yielded 2125 women.

Five years after delivery, an invitation for a follow-up study was sent to the home address of all mothers to singleton healthy children. The invitation included study information, consent form for both caregivers, a questionnaire and a pre-stamped envelope for sending back the completed documents to the research staff. All women gave their informed consent for inclusion before they participated in the study. Both parents gave consent for the participation of their child. The data collection was conducted according to the Declaration of Helsinki and the study was approved by the Regional Ethics Committee in Gothenburg, and the Swedish Ethical Review Authority (protocol code 897–11, T439-13, T085-14 and 2019–05,219).

### Data collection

During pregnancy, two venous blood samples were taken at the antenatal care clinic at routine visits; one in early pregnancy (T1, before gestational week 17), and one in late pregnancy (T3, after gestational week 31) for analysis of 25-hydroxyvitamin D (25OHD) in serum. During these two visits, the pregnant women also completed a questionnaire regarding intake of vitamin D in the diet, use of vitamin D containing supplements and background information (i.e. education level and ethnicity). Maternal dietary vitamin D intake (µg/day) in T3 was estimated from a short food frequency questionnaire [22], containing questions on frequency and quantity of four vitamin D rich foods. Maternal use of vitamin D supplement was dichotomized as yes/no in either T1 or T3. Additional information in T1 on the women’s occupational status, tobacco use and BMI was collected from medical records. After delivery, information on the child´s birth weight, sex and delivery mode (vaginal or Caesarean section) were obtained from medical records.

The follow-up questionnaire included questions concerning any experienced asthma, eczema and/or allergic reactions to food of the child between birth and follow-up as reported by the parents. The questionnaire was an adapted version of the questionnaire developed for the BAMSE cohort study [23]. Twelve common food allergens (cow’s milk protein, eggs, fish, nuts, peanuts, cashew nuts, sesame seeds, peas, soy, cereals, stone fruits and citrus fruits) were listed in the questionnaire with an option to specify other food allergens. The questionnaire queried for information regarding atopic heredity by asking both parents to report any allergies.

### Laboratory analysis

Details on the procedures for sample collection and analysis has been described in a previous publication [21]. Briefly, venous blood samples were centrifuged within two hours after sampling and sent to the central laboratory at Sahlgrenska University Hospital. Serum was extracted and stored at -70 °C until analysis of 25OHD. Analyzes of 25OHD were performed using liquid chromatography tandem-mass spectrometry (LC–MS/MS; Mass spectrometer API 4000, AB Sciex, Framingham, MA, USA) by the central laboratory at the University Hospital in Malmö, certified by the Vitamin D External Quality Assessment Scheme.

### Statistical analysis

Cumulative incidence of atopic diseases was reported. Maternal 25OHD was season-adjusted using a cosine function as previously described [21]. The associations between maternal vitamin D intake from the diet in T3, supplement intake in T1 and/or T3 or maternal 25OHD at T1 and T3 were studied in relation to asthma, eczema or food allergy during early childhood using multivariable logistic regression. Potential confounders were identified using directed acyclic graphs [24]. Maternal BMI at T1, education (university level, yes/no), ethnicity (Northern Europe, yes/no), delivery mode (vaginal or Caesarean section) and tobacco use by either mother or other care giver during pregnancy were identified as confounders and were included a priori in the adjusted models. The child’s atopic heredity (parental allergy, yes/no) was identified as a possible effect modifier. For significant effect modifiers, stratified analyses were included. Significance was accepted at p < 0.05 (p < 0.2 for interaction terms). The software IBM SPSS Statistics for Windows, version 27.0 (Armonk, NY: IBM Corp) was used for all statistical analyses.

## Results

### Participant characteristics

Characteristics of the women and their children are described in Table 1. Of the responding families at the follow-up (*N* = 606, 33%), mean maternal age was 32 years and mean BMI was 24 kg/m2 in T1. The majority had studied at university level (77%) and were born in Northern Europe (88%). Also, the majority of the women had 25OHD concentrations above 50 nmol/L both in T1 (85%) and in T3 (81%). The children’s mean birthweight was 3540 g and their mean age at follow-up was 5.14 ± 0.4 years. Compared to the non-responding mothers (*N* = 1252), the responding study population at the follow-up were older, had a lower BMI in T1 and a higher 25OHD during pregnancy. A larger proportions of the mothers had a university education and were born in Northern Europe (data not shown). However, there were no significant differences in birthweight between the responders and the non-responders.

**Table 1:** Background data of the study population (*N* = 606)

Maternal variables	Mean ± SD or N (%)
Age T1 (years)	32 ± 4.4
BMI T1 (kg/m^2^)	23.8 ± 3.5
25OHD T1 (nmol/L)	70.2 ± 20.9
25OHD T3 (nmol/L)	85.0 ± 27.2
Dietary vitamin D intake T3 (µg/day)	4.4 ± 2.2
Vitamin D supplement use T1 and/or T3 (yes)	375 (62)
University level education (yes)	467 (77)
Northern European origin (yes)	536 (88)
**Child variables**	
Sex (female)	291 (48)
Birth weight (g)	3540 ± 527
Delivery by cesarean section (yes)	84 (14)
Tobacco exposure in utero (yes)	33 (5)
Reported atopic heredity (yes)	189 (31)
Reported asthma (yes)	76 (13)
Reported eczema (yes)	133 (22)
Reported food allergy (yes, any)	111 (18)
Cow’s milk	60 (10)
Egg	26 (4)
Fish	8 (1)
Nuts	15 (3)
Peanut	13 (2)
Cashew nut	11 (2)
Pea	2 (0.5)
Soy	6 (1)
Cereals	9 (2)
Stone fruit	11 (2)
Citrus fruit	4 (1)
Other	42 (7)

*25OHD* 25-hydroxyvitamin D, *T1* early pregnancy, *T3* late pregnancy, *BMI* Body Mass Index, *SD* Standard deviation

The reported cumulative incidence of asthma was 13% (*N* = 76), and eczema was 22% (*N* = 133). Among 18% (*N* = 111) of the children, allergic reactions to at least one food group or allergen was reported and a third of them (29%, *N* = 32) had a reaction to more than one food group or allergen.

### Maternal vitamin D status and intake in relation to asthma, eczema and food allergy

There was an interaction between both maternal vitamin D intake and status with reported atopic heredity. Therefore, all results were analyzed stratified by reported atopic heredity (Tables 2–3).

**Table 2:** The relationship between maternal vitamin D intake and status with self-reported asthma at 5-years

	**No reported atopic heredity**			**Reported atopic heredity**		
	**OR**	**95% CI**	***P*-value**	**OR**	**95% CI**	***P*-value**
**T3 dietary vitamin D intake (µg/day)**	0.925	0.775–1.105	0.389	0.936	0.740–1.184	0.582
**Supplemental vitamin D intake in T1 and/or T3 (yes)**	1.095	0.567–2.114	0.787	0.739	0.284–1.920	0.535
**T1 25OHD (nmol/L)**	1.015	0.999–1.030	0.070	0.981	0.956–1.008	0.116
**T1 25OHD (nmol/L)**						
> 75 (ref)	1.0			1.0		
< 50	0.385	0.135–1.099	0.074	0.907	0.155–5.287	0.913
50–75	0.271	0.127–0.580	0.001	1.368	0.519–3.606	0.526
**T3 25OHD (nmol/L)**	1.014	1.002–1.026	0.022	0.994	0.975–1.014	0.567
**T3 25OHD (nmol/L)**						
> 75 (ref)	1.0			1.0		
< 50	0.455	0.097–2.137	0.318	0.502	0.056–4.502	0.538
50–75	0.579	0.262–1.279	0.177	1.218	0.450–3.295	0.698

*T1* early pregnancy, *T3* late pregnancy, *25OHD* 25-hydroxyvitamin D, *OR* Odds ratio, *CI* Confidence interval, *ref* reference category

Adjusted for maternal BMI in T1, level of education (university level, yes/no), ethnicity (northern Europe, yes/no), delivery mode (vaginal or Caesarean section) and tobacco use by either mother or other caregiver during pregnancy

**Table 3:** The relationship between maternal vitamin D intake and status with reported eczema at 5-years

	**No reported atopic heredity**			**Reported atopic heredity**		
	**OR**	**95% CI**	***P*-value**	**OR**	**95% CI**	***P*-value**
**T3 dietary vitamin D intake (µg/day)**	1.141	1.011–1.288	0.032	0.879	0.725–1.066	0.191
**Supplemental vitamin D intake in T1 and/or T3 (yes)**	1.367	0.803–2.329	0.250	0.580	0.278–1.211	0.147
**T1 25OHD (nmol/L)**	0.997	0.985–1.009	0.632	1.003	0.986–1.020	0.712
**T1 25OHD (nmol/L)**						
> 75 (ref)	1.0			1.0		
< 50	0.703	0.314–1.573	0.391	1.213	0.372–3.955	0.749
50–75	1.014	0.582–1.764	0.962	0.838	0.397–1.769	0.643
**T3 25OHD (nmol/L)**	1.003	0.994–1.013	0.503	0.993	0.978–1.008	0.363
**T3 25OHD (nmol/L)**						
> 75 (ref)	1.0			1.0		
< 50	0.270	0.060–1.221	0.089	0.967	0.257–3.637	0.960
50–75	1.422	0.801–2.526	0.230	1.320	0.605–2.879	0.486

*T1* early pregnancy, *T3* late pregnancy, *25OHD* 25-hydroxyvitamin D, *OR* Odds ratio, *CI* Confidence interval, *ref* reference category

Adjusted for maternal BMI in T1, level of education (university level, yes/no), ethnicity (northern Europe, yes/no), delivery mode (vaginal or Caesarean section) and tobacco use by either mother or other caregiver during pregnancy

### Asthma

Regardless of the reported atopic heredity of the child, neither maternal vitamin D intake from diet nor vitamin D supplement use was associated with asthma (Table 2). However, among children without reported atopic heredity, maternal 25OHD of 50–75 nmol/L in early pregnancy was associated with a lower odds of reported asthma, compared to maternal 25OHD > 75 nmol/L (OR 0.271, 95% CI 0.127–0.580). Also, maternal 25OHD < 50 nmol/L was non-significantly associated with reported asthma (OR 0.385, 95% CI 0.135–1.099). In late pregnancy, maternal 25OHD, as a continuous variable, was positively associated with reported asthma (OR 1.014, 95% CI 1.002–1.009). None of these associations were seen among children with reported atopic heredity.

### Eczema and food allergy

Among children without reported atopic heredity, there was a positive association between maternal dietary vitamin D intake and reported eczema (OR 1.141, 95% CI 1.011–1.288) (Table 3). Neither maternal vitamin D status nor vitamin D supplement use was significantly associated with reported eczema. Reported food allergy was not associated with either maternal dietary or supplemental vitamin D intake, or with maternal vitamin D status (see Supplementary table S1, Additional file 1).

## Discussion

The results from this prospective cohort study using reported data show that higher maternal intake of vitamin D and vitamin D status during pregnancy were associated with higher risk of reported asthma and eczema in the children, but only among those without reported atopic heredity. There were no significant associations between either vitamin D intake or status with atopic diseases in children with atopic heredity. Thereby, atopic heredity modified the association between maternal vitamin D in pregnancy and reported atopic outcomes in the children up to 5 years of age.

Our finding of a positive association between maternal 25OHD and childhood asthma among those without reported atopic heredity partly agrees with results from two previous studies. Gale et al. [10] showed that higher maternal 25OHD (> 75 nmol/L) during late pregnancy was associated with a greater risk for the child of developing asthma at age 9 years. Similarly, the Danish cohort study showed an increased risk of respiratory problems, including asthma, among adults whose mothers had higher maternal 25OHD concentrations during pregnancy [11]. To our knowledge, we are the first to report the modifying effect of reported atopic heredity on the relation between maternal vitamin D status and reported respiratory symptoms in childhood. The lack of accounting for atopic heredity may possibly explain why contradictory associations between maternal vitamin D status and asthma in childhood has been obtained in previous research.

We found also that higher dietary vitamin D intake was associated with higher risk of reported eczema, but only in children without reported atopic heredity. Possibly, this association is explained by residual confounding since we did not find a relationship between maternal 25OHD and the children’s risk of reported eczema. Some previous studies have found no association between maternal 25OHD and eczema in the child [16, 25]. In contrast, Gale et al. found a significantly increased risk of diagnosed eczema at 9 months of age in children whose mothers had high 25OHD during pregnancy [10]. Another recent study showed that maternal vitamin D levels in the highest tertile (> 46 nmol/L) were associated with an increased risk of eczema in the infant at 12 months age [26].

Possible mechanisms for the suggested association could be through the involvement of vitamin D in the pathogenesis of atopic diseases [6]. The development of the fetal immune system seems to be affected by vitamin D. It is suggested that vitamin D status in utero might potentially impact the child´s sensitization to different allergens. Vitamin D has also been found to impact the fetal lung growth and maturation in utero [27]. Therefore, maternal vitamin D status has also been suggested to affect the lung function of the fetus. Taken together, maternal vitamin D status might play a role in the development of atopic diseases in the child [6]. Our finding of effect modification of reported atopic heredity is novel. Effect modification has however been noted in intervention trials with omega-3 fatty acids during pregnancy for prevention of atopic diseases in the offspring [28, 29]. Future research should consider this possible interaction, in order to clarify the significance of the finding.

About 60% of cases of atopic eczema start in children under the age of 5 [30]. In the Swedish BAMSE cohort, the asthma prevalence was rather stable between 4 and 12 years of age [31]. We found that the proportion of children with reported eczema under the first 5 years of life was 22%, which is in line with the diagnosed prevalence of 20–30% among children in Sweden in 2018 [32]. Further, the proportion of children with reported asthma in our study (13%) is in line with the diagnosed asthma incidence of 16% in preschool children in Sweden in 2015 [33]. The proportion of children with reported allergic reactions to food in our study (18%) are similar to previously reported 21% at 8 years of age in Sweden [34].

A limitation of our study is the inability of the questionnaire used at the follow-up to differentiate between atopic and non-atopic conditions of the outcomes. In addition, all outcomes were self-reported by the parents. Thus, misclassification of atopic conditions is possible. Also, atopic heredity was based on reported parental allergy, and did not include other atopic diseases. Even though self-reported data of parental atopy are commonly used, conclusions should be made with this in mind. Our results reflect the risk of asthma, eczema and food allergy at any time point from birth to 5 years of age. Thus, there is a risk of recall bias, as the parents may have forgotten to report symptoms that occurred in the first years of the child´s life. Another limitation was the loss to follow-up and thereby presence of selection bias. The mothers who participated in the follow-up study had a higher level of education, lower BMI in T1 and higher vitamin D status during pregnancy compared to non-participants. The extent to which the selection bias has affected our results is unknown, but it may limit the external validity of our results.

A major strength of this study is that the women’s vitamin D status was measured in both early and late pregnancy. An additional strength of the study is that 25OHD was measured by a liquid chromatography method; LC–MS/MS, which is considered a good method as it usually has high specificity and reproducibility. The consideration of multiple exposures of maternal vitamin D as well as multiple atopic outcomes, and the control for several potential confounders are additional strengths. Finally, the ability to test for and stratify by reported atopic heredity was shown to be essential for the interpretation and meaning of the results. Although, the cohort design does not allow for any conclusions on causal effects of maternal vitamin D status and vitamin D intake, our results render important aspects of including reported atopic heredity when investigating these associations.

## Conclusions

This study found reported atopic heredity to be a possible effect modifier of the association between maternal vitamin D intake and status during pregnancy and the child´s risk of developing atopic disease up to 5 years of age. In children with no reported atopic heredity, higher maternal vitamin D intake and status during pregnancy increased the risk of reported atopic diseases. In children with reported atopic heredity, there were no significant associations between either vitamin D intake or status with reported atopic diseases.

## Supplementary Information

**Additional file 1:** **Supplementary table S1.** The relationship between maternal vitamin D intake and status with self-reported food allergy at 5-years.

## Data Availability

The datasets generated and/or analysed during the current study are not publicly available due to The General Data Protection Regulation under Swedish law but are available from the corresponding author on reasonable request.

## Abbreviations

25OHD: 25-Hydroxyvitamin D
T1: Early pregnancy
T3: Late pregnancy
OR: Odds ratio
BMI: Body mass index
LC–MS/MS: Liquid chromatography tandem-mass spectrometry
SD: Standard deviation
CI: Confidence interval
ref: Reference category
