# Supplementary material for: Atopic heredity modifies the association between maternal vitamin D status in pregnancy and the risk of atopic disease in childhood: an observational study
Source: Nutr J. 2022 May 17;21:32. doi: 10.1186/s12937-022-00787-9 (PMC9112577; doi:10.1186/s12937-022-00787-9)
Supplement: Supplementary file 1 — Additional file 1: Supplementary table S1. The relationship between maternal vitamin D intake and status with self-reported food allergy at 5-years. [file 12937_2022_787_MOESM1_ESM.docx]

Atopic heredity modifies the association between maternal vitamin D status in pregnancy and the risk of atopic disease in childhood: an observational study

Anna Amberntsson ^1,^*, Ellinor Carlson Kjellberg ^1^, Jenny van Odijk ^1, 2^, Andrea Mikkelsen ^1, 3^, Linnea Bärebring ^1^ and Hanna Augustin ^1^

^1^ Department of Internal Medicine and Clinical Nutrition, Sahlgrenska Academy, University of Gothenburg, Gothenburg, Sweden; ECK: ellinor.carlson.kjellberg@hotmail.com, JvO: jenny.vanodijk@nutrition.gu.se, AM: andrea.mikkelsen@gu.se, LB: Linnea.barebring@gu.se, HA: hanna.augustin@gu.se
^2^ Department of Respiratory Medicine and Allergology, Sahlgrenska University Hospital, Gothenburg, Sweden
^3^ Research and Development Primary Health Care, Gothenburg and Södra Bohuslän, Region Västra Götaland, Sweden
* Correspondence: AA: anna.amberntsson@gu.se; Tel.: +46317863705

**Supplementary table S1.** The relationship between maternal vitamin D intake and status with self-reported food allergy at 5 years of age.

|  | **No atopic heredity** | | | **Atopic heredity** | | |
| --- | --- | --- | --- | --- | --- | --- |
|  | **OR** | **95% CI** | **P** | **OR** | **95% CI** | **P** |
| **T3 dietary vitamin D intake (µg/day)** | 0.936 | 0.812-1.078 | 0.356 | 1.125 | 0.924-1.370 | 0.240 |
| **Supplemental vitamin D intake in T1 and/or T3 (yes)** | 1.180 | 0.664-2.097 | 0.573 | 0.698 | 0.303-1.606 | 0.398 |
| **T1 25OHD (nmol/L)** | 1.008 | 0.995-1.022 | 0.234 | 0.995 | 0.975-1.016 | 0.664 |
| **T1 25OHD (nmol/L)** |  |  |  |  |  |  |
| <75 (ref) | 1.0 |  |  | 1.0 |  |  |
| <50 | 0.736 | 0.304-1.782 | 0.497 | 1.870 | 0.499-7.008 | 0.353 |
| 50-75 | 1.165 | 0.628-2.161 | 0.627 | 0.854 | 0.638-1.982 | 0.713 |
| **T3 25OHD (nmol/L)** | 1.007 | 0.996-1.017 | 0.216 | 0.997 | 0.981-1.014 | 0.754 |
| **T3 25OHD (nmol/L)** |  |  |  |  |  |  |
| <75 (ref) | 1.0 |  |  | 1.0 |  |  |
| <50 | 1.153 | 0.423-3.144 | 0.781 | 1.153 | 0.423-3.144 | 0.781 |
| 50-75 | 1.133 | 0.589-2.179 | 0.708 | 1.133 | 0.589-2.179 | 0.708 |

T1, early pregnancy; T3, late pregnancy; 25OHD, 25-hydroxyvitamin D; OR, Odds ratio; CI, Confidence interval; ref, reference category

Adjusted for maternal BMI in T1, level of education (university level, yes/no), ethnicity (northern Europe, yes/no), delivery mode (vaginal or Caesarean section) and tobacco use by either mother or other caregiver during pregnancy
